# Targeting IL‐11 in the treatment of BK virus‐associated haemorrhagic cystitis—A promising new approach

**DOI:** 10.1111/jcmm.15546

**Published:** 2020-06-25

**Authors:** Laila Schneidewind, Thomas Neumann, William Krüger, Oliver Walther Hakenberg, Christian A. Schmidt

**Affiliations:** ^1^ Department of Urology University Medical Center Rostock Rostock Germany; ^2^ Department of Hematology/Oncology University Medical Center Greifswald Greifswald Germany

**Keywords:** 3D cell culture, allogeneic stem cell transplantation, BK polyomavirus (BKPyV), STAT3, Interleukin 11

## Abstract

The BK polyomavirus (BKPyV) has pathogenic relevance especially in immunocompromised patients. No causal therapy has been established yet. Therefore, new therapeutic targets need to be identified in experimental studies. A 3D organotypic cell culture model with primary urothelial cells and fibroblasts was used as infection model. The detection of virus replication was performed with quantitative polymerase chain reaction (qPCR), and immunohistochemistry (IHC) was also used for analysis. Interleukin levels were measured by enzyme‐linked immunosorbent assay (ELISA). Interestingly, the signal transducer and activator of transcription 3 (STAT3) pathway seems to be activated during infection with BKPyV, for example phosphorylated STAT3 is significantly (*P* < 0.0001) elevated on day 6 following infection. Therefore, we performed ELISAs for involved interleukins in STAT3 pathway. Interleukin 11 (IL‐11) was significantly (*P* = 0.026) elevated at day 9. Subsequently, 3D cultures were treated with IL‐11 neutralizing antibody. At day 9 following infection, the median virus replication rate is 4.4 × 10^6^ copies/ml. The difference to replication rate without treatment was significantly lower at day 6 (*P* < 0.0001) and at day 9 (*P* < 0.0001), respectively. STAT3 pathways seem to be involved during BKPyV infection and need further investigation in experimental studies. A very promising target for treatment might be IL‐11.

## 1 INTRODUCTION

The BK polyomavirus (BKPyV) can lead to opportunistic infections and reactivation in immunocompromised patients.^1^, ^2^ BK viruria occurs in 25% up to 100% of the stem cell transplanted patients and can lead to BKPyV‐associated haemorrhagic cystitis in up to 40%.^2^, ^3^ The most important point about BKPyV‐associated haemorrhagic cystitis is that it can lead to severe morbidity, and even mortality, in stem cell transplanted patients.^2^, ^4^, ^5^ Despite the fact that a BKPyV‐associated haemorrhagic cystitis can be severe and lead to patient morbidity, no causal therapy has been established yet^2^, ^5^. Particularly, because an appropriate cell culture model for archetype virus replication is missing and therefore knowledge about the viral life cycle, too.^6^

Furthermore, researchers are recognizing the limitations of two‐dimensional (2D) cell cultures, given the fact that they do not reproduce the morphology and biochemical features that the cells possess in their original tissue.^6^, ^7^ As an alternative, the three‐dimensional (3D) cell culture approach offers the possibility to study cell growth and differentiation under conditions that more closely resemble the in vivo situation with regard to cell shape and cellular environment, especially in epithelial cell cultures.^6^, ^7^, ^8^ These 3D culture models enable to study pathogen‐host interactions and can be adapted to examine viral pathogenesis and therefore identify new therapeutic targets. Additionally, novel antiviral agents for those viruses, that are not cultivable in permanent cell lines, can be tested.^6^, ^7^, ^8^

Therefore, our study group developed an organotypic 3D cell culture model of primary urothelial cells as well as fibroblasts and established an infection with archetype BKPyV in this culture. Interestingly, during description of parts of the viral life cycle, we observed that the proliferative activity in the urothelium is significantly increasing during infection with BKPyV, while the cultures are losing differentiation. Furthermore, the STAT3 (signal transducers and activators of transcription 3) pathway might be involved in this increased proliferative activity of the urothelium during infection, because found the expression of pSTAT3 (phosphorylated/activated STAT3; pTyr705‐STAT3) significantly increased on day 6 (*P* < 0.0001) and on day 9 (*P* < 0.0001) following infection.^6^

Consequently, we were raising the question which inflammatory interleukins could be involved in this activation of STAT3 pathway during infection with BKPyV, since interleukins are interesting targets for drug development. On the whole, the primary aim of this explorative experimental study was to identify interleukins which might be involved in this infection and the secondary aim was if they could be used for therapeutic purposes.

## 2 MATERIAL AND METHODS

### 2.1 3D cell culture of urothelium as infection model for archetype BKPyV and involvement of STAT3 pathway

The 3D organotypic cell culture of primary urothelial cells and primary fibroblasts was cultivated strictly to our published protocol.^6^ Furthermore, the study has been conducted according to the Declaration of Helsinki principles. On day 3 after initiation of the cell culture, we infected the primary urothelial cells with BKPyV‐WM12 (1 × 10^7^ genomic equivalents) and washed them out again, also according to our published protocol.^6^

On day 6 and day 9 following infection, 3D cultures were fixed in formalin and then paraffin‐embedded. HE staining and immunohistochemistry (IHC) of 5 µm pTyr705‐STAT3 (pSTAT3) were performed with an antibody from R&D Systems (Wiesbaden, Germany), as described by us and Walch‐Rückheim et al.^6^, ^9^, ^10^ Cultures were evaluated with standardized settings with a DMI 6000B microscope (Leica, Wetzlar, Germany) and the Microsoft Image Composite Editor program.

### 2.2 Measurement of viral replication and assessment of inflammatory interleukins

To detect virus replication, we harvested the supernatants on defined time‐points: days 0, 3, 6 and 9 following infection, respectively. Isolation of DNA from supernatants was performed using the NucliSENS® EasyMAG® (Biomerieux, Nürtingen, Germany) system. Analysis of virus replication then was carried out by quantitative real‐time PCR (qPCR) (LightMix Polyoma JC‐BK, Tib Molbiol, Germany), as we described before.^6^

The inflammatory interleukins IL‐6 and IL‐11 were measured also on defined time‐points (days 0, 3, 6 and 9 following infection) from supernatants of the 3D cell culture by enzyme‐linked immunosorbent assay (ELISA) with the DuoSet from R&D Systems (Wiesbaden, Germany) according to the supplier´s instructions. The detection limits were 0.7 pg/ml for IL‐6 and 8.0 pg/ml for IL‐11, respectively.

### 2.3 Treatment of 3D cultures with neutralizing IL‐11 antibody

The 3D cell cultures were treated with the neutralizing IL‐11 antibody MAB 218 (monoclonal) provided by R&D Systems (Wiesbaden, Germany) in the neutralization dose ND50 (5 µg/mL) in the media from day 0 following infection onwards.

### 2.4 Definitions and statistical analysis

We included 3 independent experiments with duplicates of infection (BKPyV), negative control and infected, treated culture with neutralizing IL‐11 antibody into our analysis. Consecutively, we calculated the mean of these raw data for statistical analysis.

For each numeric variable, the numeric distribution was preliminarily assessed by the Kolmogorov‐Smirnov test. Descriptive statistics were made with mean and standard deviation for normal distribution or with median and IQR for non‐parametric data. For parametric continuous variables, Student's t test was used, and for parametric categorical variables, the chi‐square test or the Fisher exact test was used. For non‐parametric data (categorical and continuous), we used the Mann‐Whitney U test. All reported p‐values were based on a two‐sided hypothesis, *P* < 0.05 was considered to be significant. All statistical calculations were performed using statistical package for the Social Sciences 24.0 software (SPSS Inc).

## 3 RESULTS

### 3.1 Validation of involvement of STAT3 pathway

We were able to reproduce our recently published results concerning the STAT3 activation during BKPyV infection in a 3D organotypic culture of urothelium. In IHC, pSTAT3 was significantly increased during infection on day 6 following infection (*P* < 0.0001) and on day 9 following infection (*P* < 0.0001), respectively.

### 3.2 Pro‐inflammatory Interleukins

On Day 0 (day of infection), we were not able to detect IL‐6 or IL‐11 in the supernatants of all 3D cultures (infection and negative control). The ELISAs of IL‐6 showed no significant differences between infection and negative control, so these results are not persuading. Interestingly, the results of IL‐11 were significant from day 6 following infection onwards (day 3 *P* = 0.073; day 6 *P* = 0.045; day 9 *P* = 0.026). Table 1 shows the exact measurements of IL‐11 in infected 3D cultures and negative controls in the supernatants in pg/ml.

**TABLE 1:** ELISA of IL‐11 in supernatants of 3D cultures in pg/ml, differences on day 3 *P* = 0.073, day 6 *P* = 0.045 and day 9 *P* = 0.026, respectively

Experiment	Day 3	Day 6	Day 9
Negative 1	877.60	343.44	605.04
Negative 2	301.48	417.20	361.59
Negative 3	415.54	393.65	538.14
Infection 1	**1264**.**08**	**931**.**48**	**3874**.**43**
Infection 2	**984**.**00**	**600**.**82**	**3280**.**52**
Infection 3	**968**.**45**	**834**.**78**	**3548**.**23**

Note

Days following infection.

### 3.3 Treatment with neutralizing IL‐11 antibody and virus replication

**FIGURE 1:**
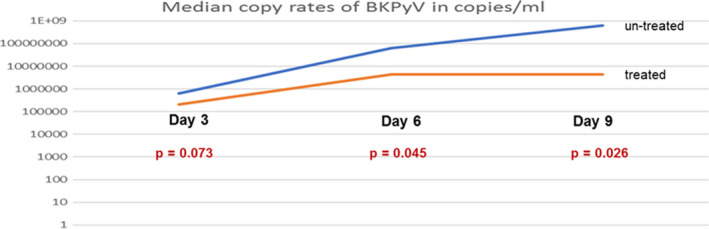
Median copy rate of BKPyV in copies/ml (qPCR) with and without treatment with IL‐11 neutralizing antibody (ND50)

Consequently, we treated the infected 3D cultures with a neutralizing IL‐11 antibody in ND50 and measured the viral replication rates by qPCR in supernatants of infected treated and untreated 3D culture as well as a negative control. At day 9 following infection, the median virus replication rate is 4.4 × 106 copies/ml (Range 4.2 × 10^6^ to 5.8 × 10^6^). The virus replication rate with treatment was significantly lower (compared to infected untreated 3D cultures) at day 3 following infection (*P* = 0.008), at day 6 (*P* < 0.0001) and at day 9 (*P* < 0.0001), respectively. Figure 1 shows the median copy rates of BKPyV in copies/mL (qPCR) with and without treatment with IL‐11 neutralizing antibody (ND50).

## 4 DISCUSSION

We conducted an explorative experimental study to prove our concept of involvement of STAT3 pathway in BKPyV infection in a 3D organotypic cell culture of urothelium and to identify interleukins which might be involved in this infection and which might be used as therapeutic targets. Interestingly, we were able to confirm our results regarding the STAT3 pathway involvement during BKPyV infection and we identified IL‐11 as interesting therapeutic target, since it was significantly elevated during infection. We initially choose to investigate STAT3 pathway, because in our first analysis we observed that during viral infection with BKPyV 3D cultures had more proliferative potential (Ki67 expression in IHC).^6^ Subsequently, we decided to treat the infected 3D cultures with a neutralizing IL‐11 antibody and so copy rates of BKPyV significantly decreased in treated cultures compared to the untreated ones. This is a novel finding and a very promising approach for a development of a treatment of BKPyV‐associated haemorrhagic cystitis.

Furthermore, it is important to develop a sufficient therapy for this disease, for example in a retrospective study of allogeneic stem cell transplantation with 2477 patients, and symptomatic BKPyV viruria was significantly associated with an impairment of kidney function and a worse overall survival.^4^ Nearly, the same was described before by Gilis et al, and the authors also put an emphasis on high financial costs to treat this complication.^5^ Additionally, in a nationwide survey of haematologists and urologists about this disease from our own study group we provided first information about plurality of applied therapies, which might be an expression of therapeutic desperation.^2^ Thereof, the development of a causal therapy and the identification of new therapeutic targets is absolutely desirable.

Despite the fact of our novel and promising finding, our experimental study has several limitations, such as the explorative nature of our study, for example we only used ND50 for our neutralization experiments. In our opinion, it is reasonable to further describe BKPyV life cycle and STAT3 pathway (upstream and downstream) in our 3D infection model to identify more interesting targets, for example other inflammatory interleukins. Furthermore, more dosing experiments for IL‐11 neutralization are warranted and our results should be validated. Due to the explorative nature of our study, we did not analyse other targets of STAT3, for example leukaemia inhibitory factor (LIF), but this might be interesting in further investigations as well.

Interestingly, the only antibody for targeting STAT3 in clinical practice is tocilizumab, which is an antibody against IL‐6 and it is used, for example in cytokine release syndrome.^11^ Despite the fact that IL‐6 showed no significant results in our experimental study, it might be reasonable to discuss the use of tocilizumab as ultima ratio for patients with severe BKPyV‐associated haemorrhagic cystitis.

On the whole, we must conclude that STAT3 pathway is involved during BKPyV infection in urothelium and it should be investigated in detail in further experimental studies to identify more therapeutic targets. IL‐11 might be a very interesting and promising therapeutic target for the treatment of BKPyV‐associated haemorrhagic cystitis, since it can be targeted with a neutralizing antibody. We plan to further evaluate this novel finding.

## CONFLICT OF INTEREST

All authors declare that they have no conflict of interest regarding this study, but this project received funding from the Monika Kutzner Foundation Berlin and Wilhelm Vaillant Foundation.

## AUTHOR CONTRIBUTION

Laila Schneidewind: Conceptualization (equal); Data curation (equal); Formal analysis (equal); Funding acquisition (lead); Methodology (lead); Resources (equal); Visualization (lead); Writing‐original draft (lead). Thomas Neumann: Data curation (equal); Formal analysis (equal); Investigation (equal); Writing‐original draft (equal); Writing‐review & editing (equal). William Hermann Krüger: Investigation (equal); Writing‐original draft (equal); Writing‐review & editing (equal). Oliver W. Hakenberg: Investigation (equal); Validation (equal); Writing‐original draft (equal); Writing‐review & editing (equal). Christian Andreas Schmidt: Conceptualization (equal); Validation (equal); Writing‐original draft (equal); Writing‐review & editing (equal).

## Data Availability

We can make the raw data available upon request.
